# Characterization of Phosphate Coatings: Influence of the Acid Pickling Conditions

**DOI:** 10.3390/ma14041048

**Published:** 2021-02-23

**Authors:** Belén Díaz, X. Ramón Nóvoa, Carmen Pérez, Sheila Silva-Fernández

**Affiliations:** CINTECX, Universidade de Vigo, ENCOMAT group, Campus universitario As Lagoas, Marcosende, 36310 Vigo, Spain; rnovoa@uvigo.es (X.R.N.); cperez@uvigo.es (C.P.); shsilva@uvigo.es (S.S.-F.)

**Keywords:** phosphate, acid pickling, citric acid, corrosion, impedance, porosity, SEM

## Abstract

This research emphasizes the importance of the acid cleaning prior to the phosphate development on high-strength steel rods. It compares the phosphate properties achieved after different acid-pickling conditions. The most common inorganic acids were considered in this study. Additionally, taking into account the environmental and safety concerns of these acids, the assessment of a less harmful organic acid is presented. This study revealed significant differences in the coating morphology and chemical composition whereas no great changes were found in terms of the coating weight or porosity. Thus, hydrochloric and sulfuric acid promote the growth of a Fe-enriched phosphate layer with a less conductive character that is not developed after the pickling with phosphoric acid. The phosphate developed after the citric acid pickling is comparable to that developed after the inorganic acids although with a porosity slightly higher. The temperature of the citric acid bath is an important parameter that affects to the phosphate appearance, composition, and porosity.

## 1. Introduction

Phosphate layers provide numerous benefits and, in spite of being a quite complex process, they are very important in many industries [1]. It is still a fundamental practice prior to the cold forming operations since it assists the sliding and provides a deposit to retain the lubricant [2]. It is a type of conversion coating where the metal substrate is replaced by an adherent layer when it is immersed in a proper bath. A more detailed explanation on the coating mechanism and specific reactions can be found elsewhere [1,2,3]. 

The importance of a properly cleaned initial surface, free from oil or scale, has been widely referred in the bibliography. In a conversion coating, a close interaction between the solution and the metal must be ensured in order to guarantee a uniform deposit. An inadequate cleaning would reduce the active sites for the coating development [1]. Thus, the effectiveness of the pre-cleaning stage plays a key role on the properties and performance of the further phosphate covering. A variety of cleaning methods have been used for this operation [4], the acid pickling being the most commonly used in the phosphating industry. It results in effective dirt removal as well as is a cost-efficient strategy. The most conventional acids for pickling are sulfuric, hydrochloric, nitric, hydrofluoric, and phosphoric acid. Their individual advantages and drawbacks have been compiled by Maanomen [5]. The specific comparison between hydrochloric and sulfuric acids has been also discussed in the technical report by Kelly [6]. 

Most of the existing literature referring to the assessment of pickling acids are essentially focused on their cleaning activity, in particular to the weight loss after the acid treatment. The importance of the variables has been recognized such as the acid concentration, the temperature, or the pickling time [1], but fewer studies were developed to compare the performance of the phosphate layers prepared after different pickling conditions. In particular, the study completed by Hivart and Bricout has evidenced the close correlation between the pickling condition and the wear performance of the phosphate layers [7]. The importance of a proper etching was also emphasized in other research areas such as the corrosion resistance of silane coatings [8] or the storage ability of supercapacitors [9]. Thus, a proper surface state improves the interaction with the involved solutions in the further stages assisting the development of an improved top layer. Other studies pointed to the importance of the etching conditions on the surface roughness that changes the wettability of the metallic surfaces [10,11].

The typical acids mentioned above have a serious disadvantage of high toxicity, since harmful gases are generated during the pickling action. In addition, the hydrogen released during the acid pickling is a serious inconvenience for high-strength steels, since it can diffuse into the metal structure contributing to hydrogen embrittlement [5]. Although the addition of pickling inhibitors could restrain this embrittlement condition, the use of this type of reagents, if done carelessly, could lead to a non-uniform surface pickling [1]. 

A feasible pickling alternative is the use of organic acids. Among them, Maanomen analyzed the efficiency of citric acid [5]. Equivalent results were obtained, at least in terms of the weight loss, when compared to hydrochloric or phosphoric acids. Its ability on removing iron oxides was also shown by Pajonk et al. [12]. One significant advantage of this acid in comparison to the usual inorganic acids is the reduced sludge formation, since Fe is dissolved in the pickling citric bath [13]. Less damage to the health of the operator along with a lower environmental impact are clear benefits of using this type of less aggressive acid pickling solution. 

This picking strategy could result in significant progress in the phosphating industry. The purpose of this research is focused to the assessment of the quality of the phosphate layers prepared after citric acid pickling. The influence of variables such as temperature and acid concentration is discussed. The study with common inorganic acids is also included to provide a realistic comparison with the most used pickling acids. 

## 2. Materials and Methods 

Hot-rolled high-strength steel bars, 11 mm in diameter, were used as the substrate, and samples about 20 mm long were cut for each test. The chemical composition is indicated in Table 1. 

Before the phosphating, the samples must be cleaned. The first stage consisted of an alkaline degreasing by immersing in NaOH 0.1 M for 5 min at ambient temperature and scrubbing with a tissue after that. The next step was the acid pickling.

The most conventional pickling acids such as sulfuric, hydrochloric, and phosphoric were tested [5,13]. The pickling with the former acid was performed at 53 ± 1 °C since it is suitable if heated. The tested acid concentrations were 196.2 g/L and 98.1 g/L and the pickling was extended for 20 min. The highest value is similar to that industrially used and the lowest one was introduced to check the effect of the acid concentration. The concentration of the hydrochloric acid was limited to 72.9 g/L, which is lower than that used in the phosphating industry. The pickling time was also fixed to 20 min to have a comparison with the sulfuric pickling. This is an acid extremely corrosive and thus the purpose was focused to validate the coating quality when using a less strong concentration. The bath was used at the room temperature to avoid an excessive gas evaporation. For the phosphoric acid, the concentration and the time were fixed to 196 g/L and 20 min, respectively, analogous to that used with sulfuric acid. The influence of the temperature was validated, in particular, in room temperature and 50 °C. It is known that the pickling efficiency of this particular acid improves significantly when heated. No inhibitors were used in order to understand as accurate as possible the impact of the several acid baths. 

As mentioned earlier, the citric acid was proposed as a less dangerous and a more environmental-friendly alternative. Two acid concentrations were tested, 96.1 and 576.4 g/L, one low concentration and one high concentration to validate the influence of the acid content. Two temperatures were checked for each concentration, 50 °C and 70 °C. Lower temperatures were not included in this study since an improved pickling efficiency was validated for temperatures in this range [5].

After the acid pickling, specimens were vigorously rinsed with tap water. Then, samples were dried with a tissue and hot air, and finally kept in a desiccator before the phosphating. 

The coating development process was initiated with a surface activation. It consisted of a 2 min immersion in a 1 g/L titanium phosphate–disodium phosphate solution at room temperature. The phosphate coatings were prepared by dipping the specimens for 6 min at 65 ± 1 °C in a bath with phosphoric acid and a Zn salt, using nitrate and nitrite as chemical accelerators [14,15,16]. The composition of the bath is included in Table 2. The pH of the solution was adjusted to 2.8 with NaOH 1 M. The bath was agitated with ultrasound to promote the development of a more densely packed structure [3]. 

The phosphate characterization was completed as follows. The coating weight was obtained with the stripping method, by measuring the weight loss after immersion for 5 min at 70 °C in a solution with Na_4_EDTA 12%, NaOH 9%, and TEA 4% [1]. From this test, the coating weight in g/m^2^, which is related to the thickness, is provided. A scanning electron microscope (SEM) was used for the structural and chemical study. A JEOL^®^ 5410 from OXFORD instruments^®^ (Abingdon, England) coupled with a Link ISIS 300 EDS detector was used. This technique allows gathering information concerning the phosphate appearance along with its composition. For the electrochemical study, the impedance spectroscopy (EIS) and the voltammetry (LSV) techniques were performed in a 0.1 M Na_2_SO_4_ solution with a potentiostat AUTOLAB PGSTAT-204 from Metrohm Autolab B.V. (Utrecht, The Netherlands). The conventional three-electrodes cell was employed with a graphite sheet as the counter-electrode and a Hg/Hg_2_SO_4_ as the reference. The electrochemical analysis consisted of a first step for the open circuit potential (OCP) stabilization for 30 min followed by the EIS measurement. The frequency range from 1 MHz to 10 mHz was scanned at the OCP with 10 mV of potential amplitude. After that, the polarization was performed, sweeping ±150 mV from the OCP in the anodic direction at 1 mV/s. These tests provide information on the coating efficiency in terms of porosity. 

## 3. Results and Discussion

### 3.1. Phosphate Coatings after Pickling with the Conventional Acids

An appropriate surface preparation is important in order to get a suitable phosphate layer. The pickling removes the scales from the specimen’s surface. The weight losses after the pickling with the sulfuric, hydrochloric, and phosphoric acids are presented in Table 3. The values were computed according to Equation (1).

(1) $$\%\;\mathrm{weight}\;\mathrm{loss}=\frac{{\mathrm{Weight}}_{\mathrm{before}\;\mathrm{pickling}}-{\mathrm{Weight}}_{\mathrm{after}\;\mathrm{pickling}}}{{\mathrm{Weight}}_{\mathrm{before}\;\mathrm{pickling}}}\times 100$$

Sulfuric acid provides the larger weight loss due to a more extended material removal. Between the two tested sulfuric acid concentrations, the highest weight loss was measured after the pickling with the lowest concentration. It seems that for the highest concentration some deposit can be accumulated on the surface during the cleaning process resulting in a reduced weight loss [7]. In fact, after rinsing with water, some black powder was found on the cloth used to wipe. Hydrochloric is an effective acid for dissolving iron oxides [7] and, according to the reduced weight loss in comparison to the sulfuric acid, a less important substrate dissolution is verified, the weight loss being mostly equivalent to the scale removal. Phosphoric acid is also less aggressive than sulfuric acid, and its efficiency is directly linked to the bath temperature. A greater weight loss may be considered as a disadvantage in terms of dimensional reduction and base metal waste.

All the pickled specimens were then subjected to the same phosphating process in order to get a comparison on the conversion coating properties according to the initial surface state. The coating weights are also indicated in Table 3. The sulfuric-pickled specimens promote the growth of heavier layers. It seems that for a poor pickling effect, that is the case of HCl or H_3_PO_4_-T_room_, a lighter phosphate is developed afterwards. However, above a certain pickling removal level, no correlation is observed between the prior metal loss and the final phosphate weight. 

**Figure 1:**
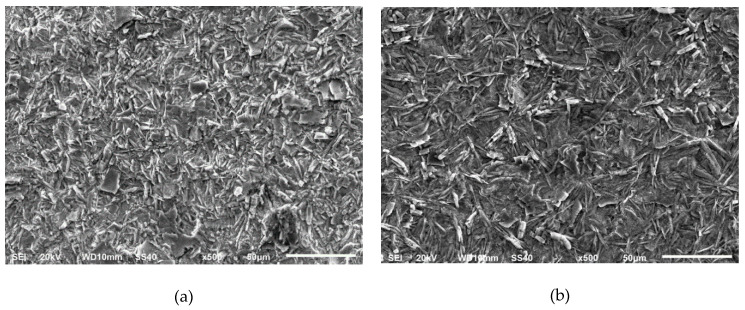

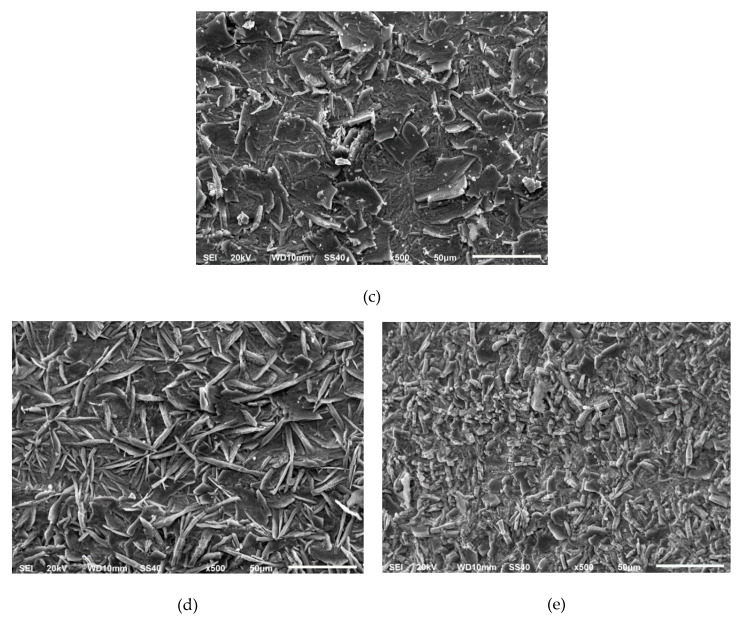
SEM images of the phosphate layers prepared after the several pickling conditions: (**a**) H_2_SO_4_ 196.2 g/L; (**b**) H_2_SO_4_ 98.1 g/L; (**c**) HCl 72.9 g/L; (**d**) H_3_PO_4_ 196.2 g/L at ambient temperature; (**e**) H_3_PO_4_ 196.2 g/L at 50 °C.

The structure and composition of the developed conversion coatings have been studied with the SEM. The SEM images are presented in Figure 1. The pictures evidence the importance of the pickling conditions on the phosphate appearance, in terms of the shape and size of the crystals.

Layers developed on the samples pickled in sulfuric acid, Figure 1a,b, are homogeneous, with a suitable coverage. They are essentially composed of thin platelets. The size of these crystals is slightly smaller when using the more concentrated acid (Figure 1a).

Phosphate layers growth after the HCl-pickling, Figure 1c, are constituted by larger crystals. A less uniform surface is provided. The number of crystals is reduced in comparison to the sulfuric pickling. This observation points to the formation of a less rough surface, with a lower number of nucleation sites, after the hydrochloric pickling in comparison to the sulfuric condition. This also agrees with the fact that the HCl majorly dissolves the oxides whereas no generalized attack on the metal substrate is produced, contrary to the sulfuric acid action [6]. 

After the pickling in the phosphoric acid at the room temperature, Figure 1d, a uniform coating with larger plates is developed. The appearance is different after the pickling at the highest temperature, Figure 1e, since a crystal refinement is detected. The crystals shape changes also to a prismatic/cubic appearance. Although a good coverage is noticed in both phosphoric-pickled specimens, the pickling at the highest temperature provides a more active surface with a large number of nucleation sites for the phosphate growth. 

The crystals show a different growth direction when the metal is pickled in phosphoric acid. Thus, they appear perpendicular to the metal substrate whereas a type of crystals parallel to the surface are developed after pickling with hydrochloric acid. A mixture of both types of crystals is observed after the sulfuric pickling.

A comparison of the chemical composition obtained from the EDS analysis is compiled in Table 4. The numbers presented are the average values taken from the whole picture analysis (×500) at different sites. The large amounts of Fe that have been recorded can be assigned to the uncovered areas and/or to the substrate response and/or to the own phosphate layer. None of these options can be ignored and then some discussion concerning this element is complex. In any case, although the precise chemical structure cannot be determined, the Zn/Fe ratio can be used to deduce some structural differences among the layers included in this discussion. The coating developed after the hydrochloric pickling contains the largest level of Fe along with the lowest content of Zn, which points to the existence of Fe-enriched phosphates such as FeZn_2_(PO_4_)_2_∙4H_2_O, Fe_3_(PO_4_)_2_, and/or FePO_4_. The layers prepared after the pickling in H_3_PO_4_ at room temperature exhibit a higher Zn content that suggests the likely development of some hopeite, Zn_3_(PO_4_)_2_∙4H_2_O, in the coating structure. The pickling in the H_3_PO_4_ at 50 °C promotes a larger Fe phosphate content. There exists a correlation between the crystal shape and the coating composition that can be deduced from the comparison between the two phosphate layers growth after pickling with phosphoric acid. Thinner plates are correlated to a higher Zn content whereas prismatic crystals point to Fe enrichment. 

The pickling efficiency (measured as the weight loss, Table 3) seems to be connected to the type of conversion coating developed afterwards. Thus, the larger the weight loss in the acid pickling, the larger the Fe content in the phosphate. By contrary, a less aggressive pickling helps in the formation of a phosphate with a lower amount of this element. 

#### Electrochemical Response of the Phosphated Specimens

**Figure 2:**
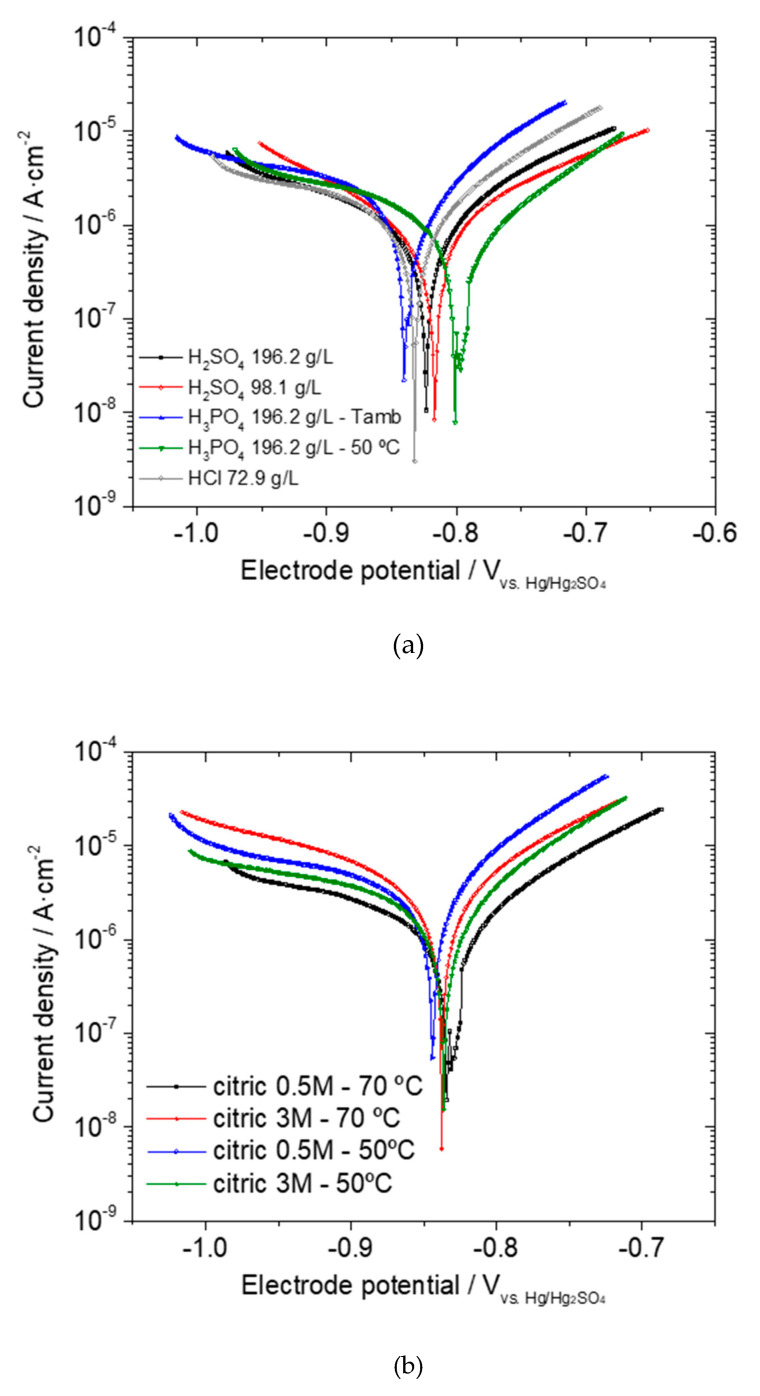
Polarization curves for the phosphated specimens obtained in a 0.1 M Na_2_SO_4_ solution: (**a**) after the pickling with the conventional acids; (**b**) after the pickling with citric acid. Potential values are referred to a Hg/Hg_2_SO_4_ electrode.

The polarization curves of the phosphate layers are compiled in Figure 2a. Table 5 summarizes the corrosion parameters deduced from the fit of the polarization curves. The results corresponding to the corrosion current density (i_corr_) are included. The porosity values obtained from Equation (2) (as percentage of uncovered surface) are also presented. No great differences were obtained among the electrochemical responses of the just pickled specimens and then the i_corr_ obtained for a specimen pickled in H_2_SO_4_ 196.2 g/L was considered as a common reference for the bare substrate condition, 20.07 µA/cm^2^.

(2) $$\%\;\mathrm{Porosity}=\frac{i_{\mathrm{corr}-\mathrm{phosphated}}}{i_{\mathrm{corr}-\mathrm{uncovered}}}\times 100$$

No relevant differences were obtained among the calculated data. For all the prepared phosphate layers, a porosity below 10% was measured, which means that the coating covers more than 90% of the specimen surface. In any case, the lowest porosity, and then the more efficient covering, were obtained for the phosphate layer prepared after the acid treatment in H_3_PO_4_ at the high temperature. By contrary, the specimen that remains with a higher percentage of uncovered surface was that previously pickled with the same acid at the lowest temperature. Apparently, there does not exist a clear correspondence between the phosphate porosity and the pickling efficiency (weight loss in Table 3). 

**Figure 3:**
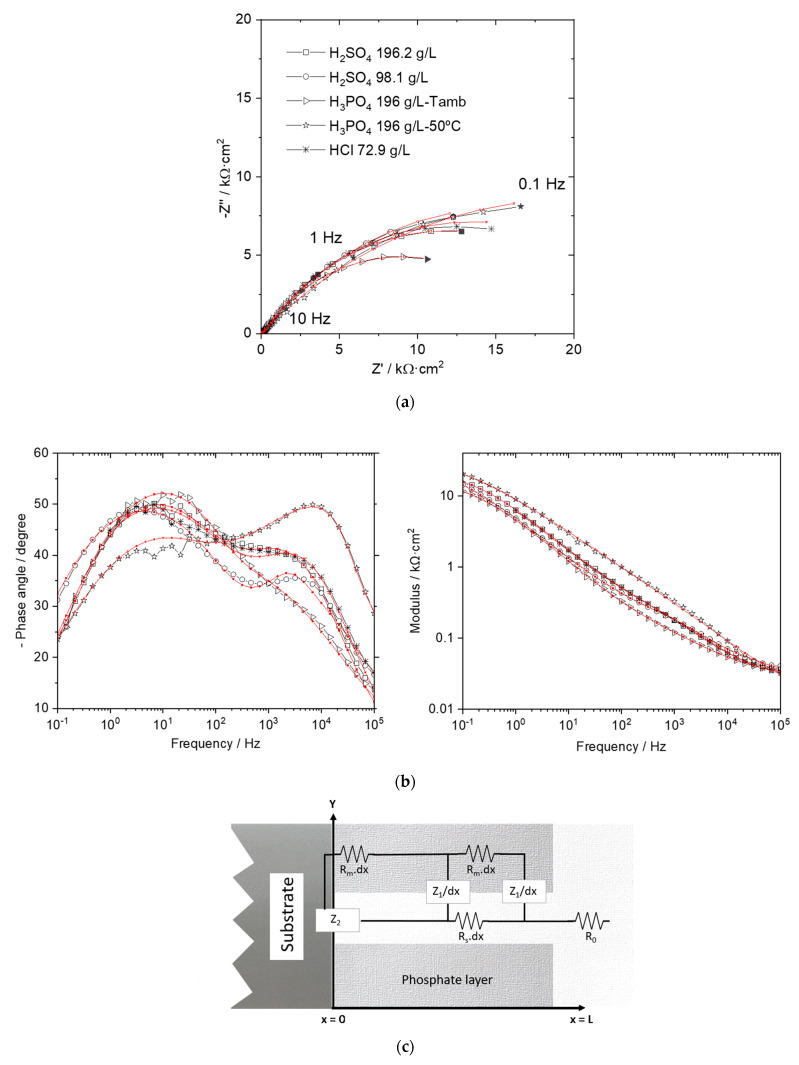
Impedance spectra obtained in 0.1 M Na_2_SO_4_ for the phosphate layers prepared after pickling with the inorganic acids: (**a**) Nyquist plots; (**b**) Bode plots; (**c**) equivalent circuit used for modelling [3,20]. Red lines denote the fitted curves.

The impedance measurements were also recorded in the 0.1 M Na_2_SO_4_ solution. Figure 3 shows the Nyquist and Bode plots obtained for the layers developed after the pickling in the usual inorganic acids. Concerning the Nyquist plots, a semicircle is observed where changes among the different pickling conditions can be hardly identified. The highest low-frequency limit value was defined for the layer prepared after the hot phosphoric pickling. The smallest one was detected after the pickling in phosphoric acid at the ambient condition. This low-frequency limit value could be assigned to the corrosion performance and the obtained plots correlate qualitatively well to those presented in Table 5. From the phase angle variation in the Bode plots, two peaks can be discerned, one in the kHz range and another in the Hz range. Intuitively, one could try to model these results with an equivalent circuit composed of two time constants, frequently used for coated systems [17,18]. Even though the corrosion behavior may be correctly assessed, unrealistic “coating capacitance” values, in the range of 10^−10^ F/cm, are obtained. These abnormal values are two orders of magnitude higher than that expected for a ceramic-type layer. Moreover, a certain conductive character was already evidenced for the phosphate layers [3] and therefore another more suitable equivalent model must be employed. 

The equivalent circuit included in Figure 3c was used to fit the impedance measurements. This model was adapted from the transmission line model originally developed by Macdonald [19]. Its suitability for the analysis of porous electroactive coatings has been verified in previous studies [3,20,21]. Besides providing the corrosion protective character through the R_2_ and C_2_ parameters (charge transfer resistance and double layer capacitance, respectively, at the bare substrate/electrolyte interface), this model has the advantage that allows the characterization of the coatings properties through the R_m_ (phosphate resistivity) and R_s_ (resistivity of the electrolyte filling the phosphate pores). The resistance R_2_ and capacitance C_2_ are arranged in parallel and represented by the Z_2_ component in the equivalent circuit of Figure 3c. In addition, the Z_1_ element that measures the interaction at the phosphate/electrolyte interface gives information on the phosphate structure. This element is defined as a parallel arrangement of a resistance R_1_ and a capacitance C_1_. The high frequency limit is defined as the electrolyte resistance, represented by the parameter R_0_. The parameters R_m_, Rs and the impedance Z_1_ are distributed along the coating thickness, which is represented by “dx.” Thus, the phosphate thickness, L, is required as a fixed value to proceed with the fitting. This length was obtained from the coating weight measurement. An average value for the phosphate density of 3 g/cm^3^ was taken to complete this calculation [1]. The fitting parameters are compiled in Table 6. A good correspondence between the experimental and the fitted data was achieved (fitted results are also incorporated in red color in Figure 3). The electrolyte resistance values are not included in Table 6 since they do not contribute to the own phosphate layer performance. Values in the range 10–24 Ω·cm^2^ were obtained. 

The high values obtained for the coating resistivity, R_m_, after the pickling with sulfuric and hydrochloric acids point to the formation of a less conductive deposit. According to the chemical composition referred in Table 2, a larger amount of Fe had been detected for these layers, in particular relevant to the HCl pickling. Thus, the development of a Fe-enriched layer with a highly insulating character is promoted, most probably containing FePO_4_ in their structure [21]. After hydrochloric and sulfuric acids pickling, the metallic surface becomes activated so that the oxidation of the Fe^2+^ ions is assisted once the phosphating process is initiated. By contrary, the phosphate growth in phosphoric acid at ambient temperature shows the lower resistivity, pointing to a larger amount of other less resistive compounds such as hopeite [22]. It should be emphasized that this layer has the highest resistance through the pores, which can be an indication of the development of a lower number of pores, that is, the growth of less (but coarser) crystals. This agrees with the coating appearance presented in Figure 1. 

Modifications concerning the Z_1_ time constant refer essentially to the C_1_ parameter. Again, the results obtained after the phosphoric pickling are completely different in comparison to the other acids. After the phosphoric pickling, the time constant is shifted to higher frequencies pointing to a faster interaction between the phosphate and the electrolyte filling the pores. This is another indication of the increased conductivity in the layer prepared after the phosphoric pickling. The low dispersion coefficients (α_1_) obtained for the layers growth after the sulfuric and hydrochloric cleaning is an evidence of a less uniform phosphate, both in composition and/or structure. The deposition of the above mentioned FePO_4_ compound on the phosphate layer may be related to the differences found in Z_1_ [3]. 

The corrosion resistance can be deduced from the R_2_ values. No great differences were revealed in this parameter, all the values being in the same order of magnitude. This is in agreement with the scarce difference also measured in the i_corr_ values (Table 5). In any case, the highest resistance and then the lowest corrosion rate was stated for the layer developed after picking in the hot phosphoric acid. The lowest value was obtained for that coating prepared after pickling in phosphoric acid at room temperature. The good agreement with the results given in Table 5 shows the suitability of the impedance fitting. The lower than expected C_2_ values found for the hydrochloric and sulfuric pickled specimens point to the contribution of another capacitance in series to the referred double layer capacitance at the bottom of the uncovered substrate. The formation of the FePO_4_ layer also on those areas could explain that variation. 

### 3.2. Phosphate Coating after Pickling with Citric Acid: Temperature and Concentration Assessment

Two citric acid concentrations and two bath temperatures were tested, with the corresponding weight losses indicated in Table 7. The influence of the temperature is evident. An increase by a factor of 5–7 was recorded in the weight loss when the temperature is raised from 50 °C to 70 °C. In the line to the results obtained from the pickling in sulfuric acid, the higher the citric concentration, the lower the mass loss. The formation of some “protective” deposit could explain the more reduced weight loss when using the more concentrated citric acid bath. An excessive hydrogen bubbles formation on the specimen could also inhibit the acid pickling activity. In terms of the workpiece dimensions, this type of acid would be preferred in comparison to the conventional inorganic acids that remove a larger portion of the material, with the exception of the tested diluted HCl or H_3_PO_4_ at room temperature. 

The phosphate layers are heavier when using the most efficient acid cleaning (Table 7), in the same trend as the results taken from the pickling with the inorganic acids (Table 3). Thus, the treatments at the highest temperature result in the development of a higher coating weight. However, the higher citric concentration, in spite of resulting in a lower weight loss after pickling, promotes the formation of a slightly thicker layer. It seems that the pickled surface remains more efficiently activated for the phosphate growth after pickling with the highest citric concentration. The precise pickling mechanism needs to be defined to understand the referred differences. The more concentrated acid could promote a more localized dissolution, and then lower weight loss, but a further study will be developed in order to clarify the surface modification along the pickling period. 

**Figure 4:**
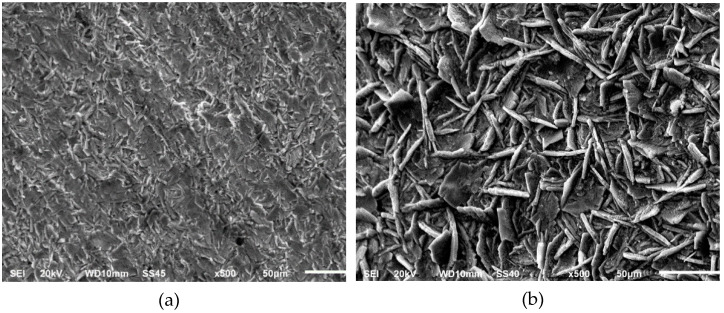

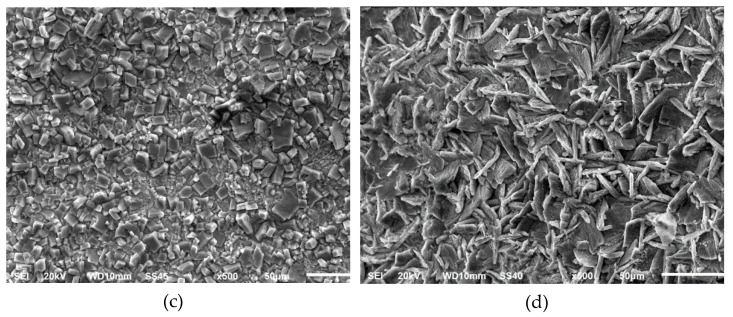
SEM images of the phosphate layers prepared after the citric acid pickling: (**a**) 96.1 g/L—50 °C; (**b**) 96.1 g/L—70 °C; (**c**) 576.4 g/L—50 °C; (**d**) 576.4 g/L—70 °C.

Figure 4 shows the SEM images of the phosphate layers developed after the citric pickling. The deposits are uniform but again their appearance is greatly influenced by the conditions of the citric acid bath. In particular, those coatings prepared after the pickling at the lowest temperature (Figure 4a,c) are constituted by tiny crystals, plate-shaped for the less concentrated pickling acid or cubic-shaped for the more concentrated pickling acid. The crystals developed after the pickling at the highest temperature (Figure 4b,d) are comparable in terms of shape (plates) and size. 

The chemical analysis is compiled in Table 8. The Fe content is largely reduced when using the highest temperature pickling bath, almost independently on the acid concentration. This observation allows a two-fold deduction. On the one hand, a lower coating weight was obtained for the less aggressive pickling (Table 7) and then a portion of this element could arise from the substrate. On the other hand, the Zn/Fe ratio is also lower for these thinner layers, which means that the as-formed phosphate layers have a lower Zn content (and larger Fe content).

After pickling at high temperature, the amount of detected Fe is significantly reduced, it is even lower than those layers prepared after the pickling with the conventional acids (Table 4). Since the coating weights are comparable to the layers obtained after the inorganic acids pickling, this difference points to a lower content of Fe in the own phosphate when grown on the citric acid pickled substrates. A further analysis must be performed to verify the influence of these structural modifications in terms of their chemical stability.

#### Electrochemical Response of the Phosphated Specimens

Table 9 compiles the corrosion rates measured from the polarization tests in the 0.1 M Na_2_SO_4_ solution and the corresponding porosity values computed according to Equation (2). The polarization curves are presented in Figure 2b. Surprisingly the thicker coating shows the highest porosity, actually it is the highest porosity value found in this study. This result evidences that the different substrate state, due to the different acid pickling, plays a relevant role in the coating growth mechanism. An increased porosity is, of course, detrimental from the corrosion point of view. However, it may be a required characteristic in cold forming operations since a porous layer allows the incorporation of the lubricant into its structure. 

**Figure 5:**
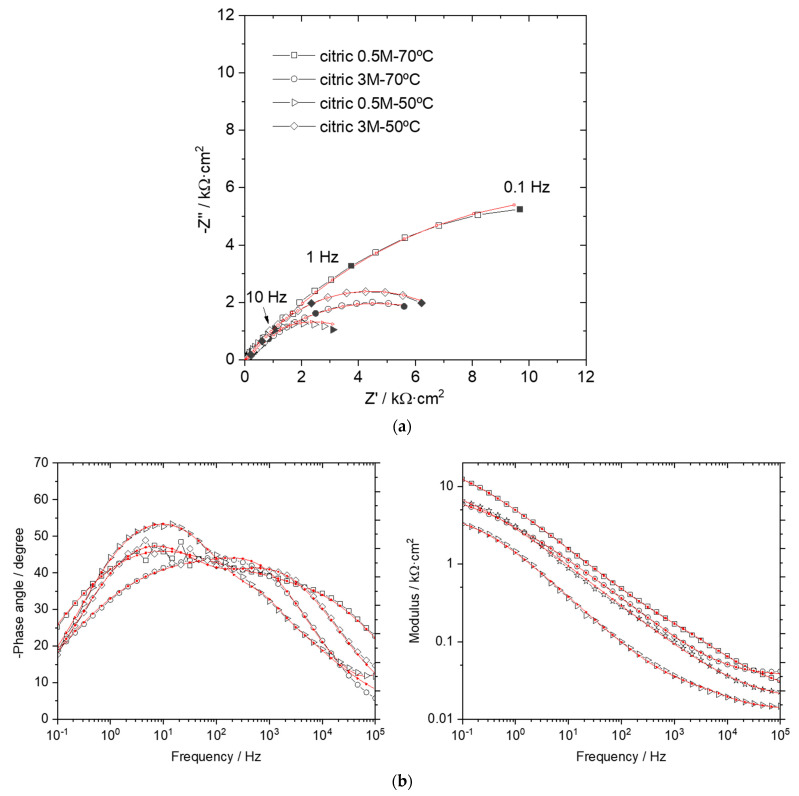
Impedance spectra obtained in 0.1 M Na_2_SO_4_ for the phosphate layers prepared after pickling with citric acid: (**a**) Nyquist plots; (**b**) Bode plots. Red lines denote the fitted curves.

Figure 5 shows the impedance spectra recorded for the layers developed after the citric acid pickling. The appearance of the spectra is similar to that shown in Figure 3 and the data were fitted following the same model as above presented (Figure 3c). The importance of a proper model choice becomes relevant for this set of results. Thus, a wrong deduction on the corrosion performance could be inferred from the plain graphical analysis. In particular, the diameter of the Nyquist semicircle does not correlate to the corrosion properties as deduced from the polarization experiments (Table 9). Then, the correct circuit must be able to provide equivalent information in terms of the layers efficiency.

The fitted values are indicated in Table 10. Some differences are evidenced and the phosphate layers can be classified in two categories, and the temperature in the cleaning acid bath being the common factor. Thus, those coatings developed after the pickling in the lowest temperature bath (both concentrations) show the highest resistivity values in the electrolyte filling the pores (R_s_). This characteristic means that the number of pores in these layers, regardless of their diameter, is lower. In fact, the SEM images show a kind of denser coating when it is grown after the pickling at the lowest temperature (Figure 4a,c). Higher values were also obtained for the coating resistivity, R_m_, which points to the likely development of the FePO_4_ insulating compound. The higher level of Fe found for these coatings (Table 7) agrees to this statement. 

The corrosion performance obtained from the R_2_ parameter shows that, according to the i_corr_ results, the coatings with the higher percentage of bare substrate exposed to the electrolyte are that developed after pickling at the highest temperature and highest concentration and that obtained after pickling at the lowest temperature and lowest concentration. Additionally, the porosity may have some influence on the R_m_ value. Thus, the two coatings grown after the pickling in the highest temperature have an analogous composition, and then the R_m_ values should be equivalent. However, due to the higher porosity developed after the pickling with the more concentrated acid, the phosphate resistivity is slightly reduced. The same result can be deduced from the comparison between the two layers prepared after pickling at the lowest temperature. 

No relevant modifications are obtained for the parameters assigned to the Z_1_ time constant. It is worth mentioning that the values obtained after the highest temperature pickling are in the same order to those obtained after the room temperature phosphoric pickling which suggests an analogous phosphate structure and composition. The similarity among the obtained SEM images (Figure 3d and Figure 4b,d) corroborates this result.

## 4. Conclusions

This study has shown the importance of the pickling stage in the process of development of phosphate layers. The acid pickling condition affects the weight loss prior to the phosphate development but also induces modifications in the characteristics of the layer. Among the inorganic acids included in this research, no differences were revealed in terms of the covering efficiency. The thickest layers are achieved when using sulfuric acid and a correlation was found between the pickling weight loss and the coating thickness. The hot phosphoric acid promotes the development of a coating with a higher Zn content. An insulating layer of Fe-phosphate is grown after pickling with sulfuric and hydrochloric acids.

Citric acid as a less damaging pickling alternative was checked as well. The coating weights are similar to those obtained for the conventional acids and the composition and appearance are analogous to those layers developed after the phosphoric pickling. Layers that are more porous are developed after the citric acid pickling but this characteristic could be beneficial for a further cold forming operation.

## Figures and Tables

**Table 1:** Chemical composition (% weight) of the steel rods used in this study.

C (%)	Mn (%)	Si (%)	S (%)	P (%)	Cr (%)	Ni (%)	Mo (%)	Cu (%)	V (%)	N (%)
0.815	0.783	0.160	0.003	0.007	0.150	0.070	0.013	0.116	0.004	0.007

**Table 2:** Phosphating bath composition (for 1 L).

H_3_PO_4_ (85 wt.%)	ZnO	NaNO_3_	NaNO_2_
8.6 mL	1.3 g	3 g	0.1 g

**Table 3:** Weight loss (%) after the pickling with the pickling acids and weights (g/m^2^) of the phosphate coatings developed afterwards.

	H_2_SO_4_ 196.2 g/L	H_2_SO_4_ 98.1 g/L	HCl 72.9 g/L	H_3_PO_4_ 196 g/L-T_room_	H_3_PO_4_ 196 g/L-50 °C
Weight loss (%, after pickling)	2.28	2.83	0.01	0.06	1.17
Coating weight (g/m^2^, after phosphating)	7.80	5.69	3.13	3.32	5.30

**Table 4:** Quantitative EDS analysis (atomic%) of the phosphate layers obtained after the several pickling conditions.

	H_2_SO_4_ 196.2 g/L	H_2_SO_4_ 98.1 g/L	HCl 72.9 g/L	H_3_PO_4_ 196 g/L-T_amb_	H_3_PO_4_ 196 g/L-50 °C
Zn	26.1	25.2	23.8	28.7	24.7
P	24.7	24.7	20.2	24.1	27.5
Fe	49.1	50.1	56.0	47.2	47.8

**Table 5:** Average corrosion data obtained from the polarization curves performed in 0.1 M Na_2_SO_4_.

	H_2_SO_4_ 196.2 g/L	H_2_SO_4_ 98.1 g/L	HCl 72.9 g/L	H_3_PO_4_ 196 g/L-T_amb_	H_3_PO_4_ 196 g/L-50 °C
i_corr-phosphated_ (µA/cm^2^)	1.00	1.29	1.15	1.77	0.88
Porosity (%)	5.01	6.40	5.73	8.8	4.39

**Table 6:** Fitting parameters obtained from the impedance recorded in 0.1 M Na_2_SO_4_ for the phosphate coatings developed after the pickling in the conventional inorganic acids.

	R_m_ (kΩ·cm)	R_s_ (kΩ·cm)	R_1_ (Ω·cm^3^)	C_1_ (mF·cm^−3^)	α_1_	R_2_ (kΩ·cm^2^)	C_2_ (µF·cm^−2^)	α_2_
H_2_SO_4_-196.2 g/L	2612.4	128.1	19.9	556.0	0.6	45.6	0.8	0.8
H_2_SO_4_-98.1 g/L	4111.9	122.1	23.1	505.9	0.6	31.2	2.1	0.8
HCl-72.9 g/L	9656.4	79.8	8	659.1	0.6	37.9	3.6	0.7
H_3_PO_4_-196 g/L-T_room_	11.3	1331.2	15.1	23.4	1	22.0	68.3	0.6
H_3_PO_4_-196 g/L-50 °C	933.1	14.9	22.6	0.2	1	68.6	100.1	0.5

**Table 7:** Weight loss (%) after the pickling with citric acid and coating weights (g/m^2^) of the phosphate developed afterwards.

	96.1 g/L		576.4 g/L	
	50 °C	70 °C	50 °C	70 °C
Weight loss (%, after pickling)	0.12	0.87	0.09	0.46
Coating weight (g/m^2^, after phosphating)	3.71	5.23	3.53	6.64

**Table 8:** Quantitative EDS analysis (atomic%) of the phosphate layers obtained after the pickling in citric acid.

	96.1 g/L		576.4 g/L	
	50 °C	70 °C	50 °C	70 °C
Zn	19.4	39.6	27.3	39.9
P	18.1	32.3	28.9	34.7
Fe	62.4	28.1	43.9	25.4

**Table 9:** Average corrosion data obtained from the polarization curves performed in 0.1 M Na_2_SO_4_.

	96.1 g/L		576.4 g/L	
	50 °C	70 °C	50 °C	70 °C
i_corr-phosphated_ (µA/cm^2^)	3.25	1.62	1.69	3.68
Porosity (%)	16.2	8.1	8.4	18.3

**Table 10:** Fitting parameters obtained from the impedance recorded in Na_2_SO_4_ 0.1 M for the phosphate coatings developed after the pickling in citric acid.

	R_m_ (kΩ·cm)	R_s_ (kΩ·cm)	R_1_ (Ω·cm^3^)	C_1_ (mF·cm^−3^)	α_1_	R_2_ (kΩ·cm^2^)	C_2_ (µF·cm^−2^)	α_2_
**50 °C—96.1 g/L**	64.1	1234.2	0.5	25.4	0.9	13.2	36.9	0.6
**70 °C—96.1 g/L**	39.7	846.1	22.2	4.6	1	29.7	104.1	0.6
**50 °C—576.4 g/L**	217.8	1557.5	1.1	6.2	0.9	37.5	27.7	0.6
**70 °C—576.4 g/L**	15.8	756.1	137.1	14.9	1	10.5	128.3	0.5

## Data Availability

Data is contained within the article or supplementary material.
